# 63. Impact of Infectious Disease Fellow-Driven Antimicrobial Stewardship Interventions on Inpatient Fluoroquinolone Use

**DOI:** 10.1093/ofid/ofab466.265

**Published:** 2021-12-04

**Authors:** Carlos M Nunez, Arun Mattappallil, Katie A McCrink, Debbie Rybak, Basil Taha, Debra Chew

**Affiliations:** 1 Rutgers NJMS, Princeton, New Jersey; 2 University Hospital, Newark, NJ, Newark, New Jersey; 3 Jackson Health System, Madison, New Jersey; 4 Crossroads Medical Group, Hillside, New Jersey; 5 Rutgers New Jersey Medical School, Newark, New Jersey

## Abstract

**Background:**

Fluoroquinolone (FQ) antibiotics are frequently used in hospitalized patients to treat a wide range of infections but are often misused and implicated in antibiotic-associated adverse events. The purpose of this study is to evaluate the impact of Infectious Disease fellow (IDF)-driven antimicrobial stewardship program (ASP) interventions on inpatient FQ use.

**Methods:**

This is a retrospective study of all admitted patients who received a FQ for greater than 48 hours from 01/01/2019 -12/31/2020 in an urban academic center. “Phase 1” (pre-intervention phase) covered 01/1/2019- 03/31/2019. “Phase 2” (intervention phase) covered 03/03/2020- 12/23/2020. In “Phase 2”, our ASP reviewed FQ use 2-3 days per week and an IDF provided feedback interventions that averaged 30-60 minutes of IDF time spent per day. We categorized FQ use as either: “appropriate”, “appropriate but not preferred”, or “inappropriate”, as determined by local clinical guidelines and ASP team opinion. We compared FQ use in both phases, indications for FQ use, and new *Clostridioides difficile* infections (CDI).

**Results:**

A total of 386 patients are included (76 in “Phase 1”and 310 in “Phase 2”). Patient characteristics are similar (Table 1). Overall, 63 % of FQ use was empiric, and 50% FQ use was deemed “appropriate”, 28% “appropriate but not preferred”, and 22% “inappropriate”. In “Phase 2”, 126 interventions were conducted, with 86% of these accepted. Appropriate FQ use increased significantly in “Phase 2” vs. “Phase 1” (53.5% vs 35.5%, p = 0.008), with decrease in mean days of FQ use (4.38 days vs 5.87 days, p =.021). Table 2 shows “appropriate” FQ use by clinical indication. New CDIs occurred more in “Phase 1” vs. “Phase 2” (6.6% vs 0.6%, p=.001).

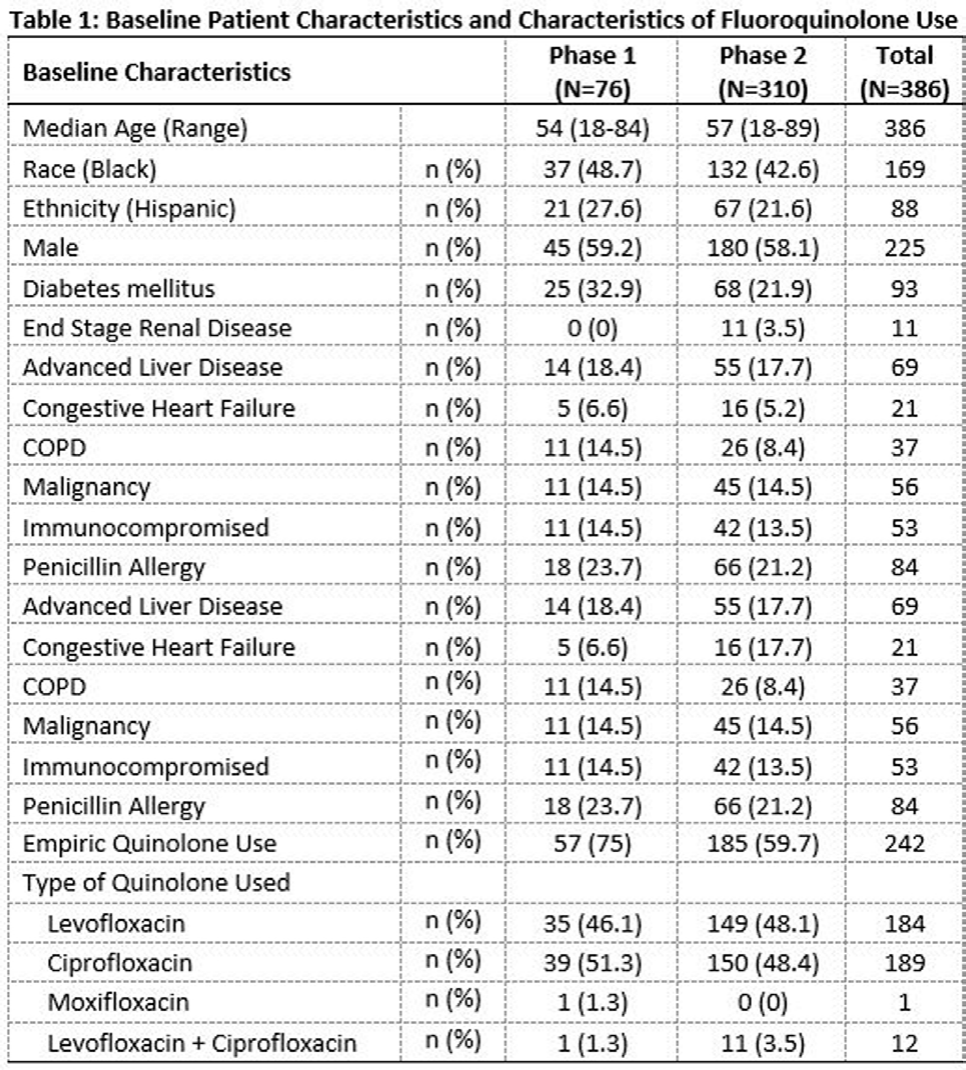

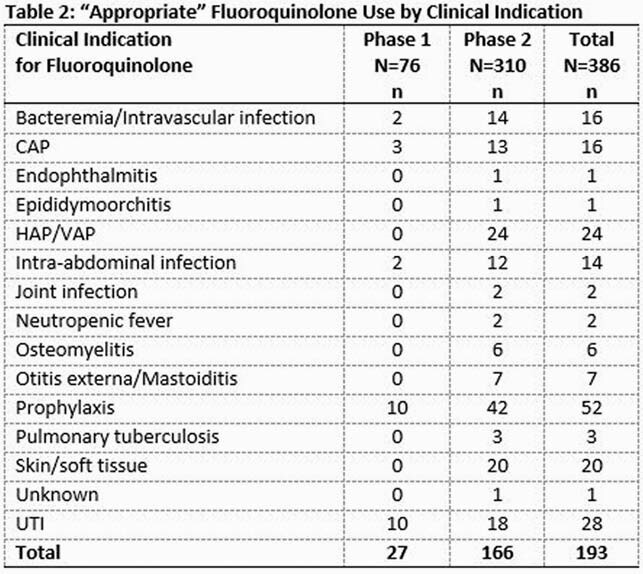

**Conclusion:**

An IDF-driven ASP intervention has a positive impact on appropriate inpatient use of FQs in our hospital. This highlights a promising ASP model which not only improves appropriate use of FQ, but also offers an opportunity for IDF mentorship and use of available resources to promote ASPs.

**Disclosures:**

**Katie A. McCrink, PharmD**, **ViiV Healthcare** (Employee)

